# *Moringa oleifera L*. Extracts as Bioactive Ingredients That Increase Safety of Body Wash Cosmetics

**DOI:** 10.1155/2020/8197902

**Published:** 2020-07-01

**Authors:** Zofia Nizioł-Łukaszewska, Dominika Furman-Toczek, Tomasz Bujak, Tomasz Wasilewski, Zofia Hordyjewicz-Baran

**Affiliations:** ^1^Department of Technology of Cosmetic and Pharmaceutical Products, The University of Information Technology and Management in Rzeszow, Kielnarowa 386a, 36-020 Tyczyn, Rzeszów, Poland; ^2^Department of Chemistry, University of Technology and Humanities in Radom, Chrobrego 27, Radom 26-600, Poland; ^3^ŁUKASIEWICZ Research Network-Institute of Heavy Organic Synthesis “Blachownia”, Energetykow 9, 47-225 Kedzierzyn-Kozle, Poland

## Abstract

The work attempts to obtain a multifunctional plant extract derived from Moringa tree leaves. Obtained results indicate a strong antioxidant potential of the tested extracts. It was shown that *Moringa oleifera* leaf extract is a rich source of flavonoid and phenolic compounds. Furthermore, it shows a strong antioxidant activity by scavenging free radicals. *In vitro* toxicity studies showed that the tested extracts in concentrations up to 5% showed a positive effect on cell proliferation and metabolism and may contribute to the reduction of oxidative stress in cells. It was noted that the tested model formulation of cosmetic (1% SCS) with the addition of different types of extracts might contribute to the reduction of skin irritation and improve the safety of the product.

## 1. Introduction

In recent years, the cosmetic industry was one of the fastest growing industries in the world. Strong competition on the cosmetic market and high consumer expectations force manufacturers to look for innovative solutions in every aspect of the product life cycle. Until recently, cosmetic manufacturers obtained the innovative advantage of their products by incorporating new raw materials and ingredients that were less common and not used by the competition. Examples include substances such as hyaluronic acid, peptides, polysaccharides, exotic oils and plant extracts, and snail slime. With time, however, these raw materials became very popular in cosmetic manufacturing and the solutions became outdated. The innovativeness of cosmetics can also be generated through the form in which skincare and beauty products are offered. Increasingly, novel forms such as foams, jellies, creams, or essences are commercially available. However, after the launch of cosmetics in innovative forms, the market tends to be almost flooded by products of the same type offered by various manufacturers. Consequently, the innovative advantage of such solutions is short-lived [1–5].

In the last few years, there has been a new trend in the cosmetic market, involving the formulation of innovative products on the basis of multifunctional ingredients. These types of substances are characterized by multidirectional activity, combining biologically active properties with moisturizing effects and the ability to give cosmetics an appropriate form or improve their safety to people and the environment. The last of the abovementioned properties are particularly sought after by present-day consumers. The strong trend for “naturalness” in cosmetics has contributed to an increase in consumer awareness with regard to substances used in cosmetic production. Consumers are looking for products which—in addition to delivering the desired usually multifaceted activity—are safe for people and the environment and are able to reduce adverse environmental impacts on the skin (antismog, antipollution cosmetics). Examples of such ingredients include plant extracts that, as has been shown in many studies, can be used as multifunctional cosmetic raw materials with moisturizing, soothing, antiwrinkle, and antioxidant properties and minimizing the adverse effects of other ingredients of the cosmetic product on the skin. The above characteristics result from the complex chemical composition of plant extracts that represent solutions of active substances derived from plants in a suitable solvent [6–13].

In the course of research in this area, much attention has been focused on the Moringa tree (*Moringa oleifera L*.), also called the tree of life, as a source of active ingredients valuable for the cosmetic industry. Due to the presence of a broad spectrum of bioactive compounds, the plant has powerful antioxidant, antibacterial, toning, astringent, and anti-inflammatory properties [14–23]. Leaves of the Moringa tree have been found to contain flavonoids including myricetin, quercetin, kaempferol, isorhamnetin, or rutin, as well as phenolic acids. Fresh leaves are a good source of carotenoids such as lutein, *β*-carotene, and zeaxanthin. In addition, the Moringa tree is characterized by a high content of vitamins C and A. The active substances contained in the plant have shown to have beneficial effects on human skin and successfully replace the synthetic ingredients [20–27].

The present study was an attempt to evaluate the effect of *Moringa oleifera* leaf extracts on the irritant potential of body wash gels. For the purpose of the study, a technology for obtaining extracts in the process of solvent extraction was developed. Water and mixtures of water with glycerin were used as natural extraction solvents. The extracts obtained this way were used for further analysis to determine their basic biochemical properties: the ability to neutralize free radicals and the content of polyphenols and flavonoids. Analyses of cytotoxicity as well as the intracellular level of reactive oxygen species were performed on *in vitro* model: keratinocytes (HaCaT) and fibroblasts (BJ) cell lines. In addition, the model cosmetics with applied extracts were also analyzed on cell lines.

## 2. Materials and Methods

### 2.1. Extract Derivation Method

Plant material was obtained commercially from Dary Natury—a Polish producer and distributor of herbs. Moringa tree leaves (*Moringa oleifera L.*) were collected on controlled and ecological crops in India and dried with warm air at 40°C for two days. The plant material has been confirmed by the distributor and Dr. Zofia Nizioł Łukaszewska (specializing in pharmacognosy). A voucher specimen of *Moringa oleifera L.* is stored in the Department of Technology of Cosmetic and Pharmaceutical Products (University of Information Technology and Management in Rzeszow, Poland). Extraction of bioactive compounds from dried leaves was carried out using the ultrasound-assisted extraction method. 5 g of grounded plant material and 100 mL of water with glycerin in various proportions (50/50, 60/40, and 80/20) were used to obtain the extract. Extraction was carried out for 10 cycles of 10 min at room temperature. Then, the obtained extracts were collected and filtered through Whatman filter paper No. 10. The material was stored in the dark at 4°C for further analysis.

### 2.2. Identification of Active Component

ESI-MS detection was performed in a 4000 QTRAP Mass spectrometer (Sciex, USA) equipped with an electrospray ionization source (ESI) and a triple quadrupole-ion trap mass analyzer that was controlled by the Analyst 1.5 software. ESI worked in the negative ion mode at the following conditions: curtain gas at 20 psi, nebulizer gas at 10 psi, and negative ionization mode source voltage−4500 V. Nitrogen was used as curtain and collision gas. MS^2^ fragmentation was used to confirm the structure of the detected compounds. The extracts of *Moringa oleifera* were diluted 100x with methanol and infused directly with a rate of 10 *µ*l/min via a syringe pump to a mass spectrometer (ESI-MS) and scanned from *m*/*z* 50 to 1000 Da under negative ion mode. High-performance liquid chromatography with tandem mass spectrometry (HPLC-ESI-MS) was used for the study of *Moringa oleifera* extracts. HPLC separation was performed on Dionex UltiMate 3000 RS Chromatograph system with computer program Chromeleon version 6.80. The eluent was monitored by electrospray ion mass spectrometer (ESI-MS) under negative ion mode and scanned from *m*/*z* 50 to 1000. HPLC separation was performed on a Kinetex 3.5 *µ*m XB-C18 100 Å column provided by Phenomenex. The mobile phases were composed of eluents A and B in gradient, where A was 0.1% (v/v) formic acid in water and B was methanol, with a flow rate of 0.6 ml/min. The elution conditions applied were as follows: 0.0–30.0 min 25–100% B, 30.0–35.0 min 100% B, 35.0–35.1 min 100–25% B, and 35.1–40.0 min 25% B. The temperature of the column was 30°C. The eluent was monitored by electrospray ion mass spectrometer (ESI-MS) under negative ion mode and scanned from *m*/*z* 20 to 1000 Da. Data analysis was processed with Analyst 1.5.1 software. The identification of selected compounds was done by molecular mass and fragment of anion entries of each individual compound and confirmed by MS^2^ fragmentation. The identities of 14 compounds were determined along with their chemical formula, deprotonated molecular ions, and the characteristic fragment ions for each individual peaks.

### 2.3. Total Phenolic Content Determination

The total phenolic content of *Moringa oleifera L.* leaf extracts was determined spectrophotometrically by the Folin–Ciocalteu method according to the procedure reported by Singleton et al. with some modifications [28]. 300 *μ*L of leaf extract solutions and 1500 *μ*L of 1 : 10 Folin–Ciocalteu reagent were mixed, and after 6 minutes in the dark, 1200 *μ*L of sodium carbonate (7.5%) was added. After 2 h of incubation in the dark at room temperature, the absorbance at 740 nm was measured spectrophotometrically by Aquamate Helion (Thermo Scientific). The total phenolic concentration was calculated from a gallic acid (GA) calibration curve (10–100 mg L^−1^). Data were expressed as gallic acid equivalents (GA)·g^−1^ of extract averaged from three measurements.

### 2.4. Total Flavonoid Content Determination

The total flavonoid content of plant extracts was evaluated using aluminium nitrate nonahydrate according to the procedure reported by Woisky and Salatino with modifications [29]. 600 *μ*L of plant extract solutions and 2400 *μ*L of mixture (80% C_2_H_5_OH, 10% Al (NO_3_)_3_ × 9 H_2_O, and 1 M C_2_H_3_KO_2_) were mixed. After 40 min of incubation at room temperature, the absorbance at 415 nm was measured spectrophotometrically by Aquamate Helion (Thermo Scientific). The total flavonoid concentration in extracts was calculated from a quercetin hydrate (Qu) calibration curve (10–100 mg·mL^−1^) and expressed as quercetin equivalents (Qu)·g^−1^ of extract averaged from three independent measurements.

### 2.5. DPPH Radical Scavenging Assay

Antioxidant activity of the plant extract was analyzed using DPPH free radical scavenging assay, according to the method described in [30]. 167 *µ*L of 4 mM ethanol solution of DPPH was mixed with 33 *µ*L analyzed samples in different concentrations (250 *µ*g·ml^−1^–5000 *µ*g·ml^−1^). The absorbance was measured at *λ* = 516 nm every 5 minutes for 30 minutes using UV-Vis spectrophotometer FilterMax 5 (Thermo Scientific). DPPH solution mixed with an equal volume of distilled water was served as a control. The percentage of the DPPH radical scavenging was calculated using the following equation:

(1) $$\begin{array}{c} \mathrm{\%DPPH}\cdot \mathrm{scavenging=}\frac{\left[Ab_{S_{\mathrm{control}}}-Ab_{S_{\mathrm{sample}}}\right]}{Ab_{S_{\mathrm{control}}}}\times 100\%. \end{array}$$

### 2.6. Zein Test

The irritant potential of the products (1% SCS with different concentrations of the extracts) was measured using the zein test [10–12]. 40 mL of the sample solution with 2 ± 0.05 g of zein from corn was shaken on a shaker with a water bath (60 min at 35°C). Then, the solutions were filtered on Whatman No. 1 filters and centrifuged at 6720*g* for 10 min. The nitrogen content in the solutions was determined by the Kjeldahl method. 1 mL of the centrifuged filtrate was mineralized in sulfuric acid (98%) containing copper sulfate pentahydrate and potassium sulfate. The mineralized solution was transferred (with 50 mL of Milli-Q water) into the flask of the Wagner–Parnas apparatus. 20 mL of 25 wt% sodium hydroxide was added. The released ammonia was distilled with steam. Ammonia was bound by sulfuric acid (5 mL of 0.1 N H_2_SO_4_) in the receiver of the Wagner–Parnas apparatus. The unbound sulfuric acid was titrated with 0.1 N sodium hydroxide. Tashiro solution was used as an indicator. The zein number (ZN) was calculated from the following equation:

(2) $$\begin{array}{c} \mathrm{ZN}=\left(10-V1\right)\times 100\times 0.7\left(\frac{\mathrm{mgN}}{\mathrm{100}\;\mathrm{mL}}\right), \end{array}$$

where *V*1 is the volume (cm^3^) of sodium hydroxide used for titration of the sample. The final result was the arithmetic mean of five independent measurements.

### 2.7. Cell Culture

HaCaT (ATCC, normal human keratinocytes) and BJ fibroblasts (ATCC CRL-2522™) were obtained from the American Type Culture Collection (Manassas, VA 20108, USA). HaCaT cells were maintained in Dulbecco's modified essential medium (DMEM, Gibco) with L-glutamine (L-g). DMEM with L-g was supplemented with 5% (vol/vol) fetal bovine serum (FBS, Gibco) and 1% (vol/vol) antibiotics (100 U·mL^−1^ penicillin and 1000 *µ*g·mL^−1^ streptomycin, Gibco). Fibroblasts were maintained in Minimum Essential Medium (MEM, Gibco) containing L-glutamine and Earle's salt, supplemented with 5% (vol/vol) FBS (Gibco) and 1% (vol/vol) antibiotics (100 U mL^−1^ penicillin and 1000 *µ*g mL^−1^ streptomycin, Gibco). All cultured cells were kept in a humidified atmosphere (95% air and 5% of carbon dioxide (CO_2_)) at 37°C. When the cells reached confluence, the culture medium was removed from the flask (VWR) and cells were rinsed two times with sterile phosphate-buffered saline (PBS, Gibco). The confluent layer was trypsinised using trypsin/EDTA (Gibco) and then resuspended in fresh medium [31, 32].

### 2.8. Cell Viability Assay

The resazurin sodium salt reduction assay was used to assess cell viability. The assay was performed according to Ivanov et al. with some modifications [33]. Cells were placed in 96-well plates at a density of 1 × 10^4^ cells/well with fresh medium. After 24 h of preculture, the medium was aspirated and various concentrations (10, 5, 3, and 1%) of tested extracts were added into each well and cultured for another 24 h. The control group was nontreated cells. After the time of exposure, resazurin salt solution (Sigma, R7017) was transferred into the plates for a final volume of 250 *µ*L/well and a concentration of 60 *µ*M in medium and incubated for 3 h at 37°C in darkness. The absorbance was measured at the wavelength *λ* = 570 nm using a microplate reader FilterMax F5 (Thermo Fisher Scientific). The experiments were performed in triplicate for each extract concentration and presented as a percentage of control values.

To evaluate if model cosmetic formulation (1% SCS) containing various concentrations (10, 5, 3, and 1%) of *Moringa oleifera* leaf extract has an effect on cell viability, the resazurin sodium salt reduction assay was used. BJ fibroblasts and keratinocytes were seeded in transparent 96-well plates at a density of 1 × 10^4^ cells/well with fresh medium. After 24 h of preincubation, cells were exposed to 30 min of model cosmetic with different concentrations of Moringa tree leaf extract. The control group was nontreated cells . After the time of exposure, resazurin salt solution (Sigma, R7017) was transferred into the wells for a final volume of 250 *µ*L/well and a concentration of 60 *µ*M in medium and incubated for 3 h at 37°C in darkness. The absorbance was measured at the wavelength *λ* = 570 nm using a microplate reader (FilterMax F5, Molecular Devices). The experiments were performed in triplicate for each tested substance concentration and presented as a percentage of control values.

### 2.9. Measurement of DCF Fluorescence

To measure the intracellular level of reactive oxygen species in HaCaT and fibroblasts cells, the fluorogenic dye H_2_DCFDA was used. After passive diffusion into the cells, H_2_DCFDA was deacetylated by intracellular esterases into the nonfluorescent compound that upon oxidation by ROS is converted to the highly fluorescent 2′, 7′-dichlorofluorescein (DCF) [34]. The fluorescence was measured according to the protocol previously described in [35]. Cells were seeded in 96-well plates at a density of 1 × 10^4^ cells per well and initially cultured before the experiment for 24 h. After this, the culture medium was changed on 10 *µ*M H_2_DCFDA (Sigma) in serum-free medium (DMEM or MEM for HaCaT and fibroblasts, respectively). Cells were incubated with H_2_DCFDA for 45 min before treatment. After this time, intracellular oxidative stress as well as production of ROS was induced by the addition of hydrogen peroxide (H_2_O_2_) to the cells at a final concentration of 1 mM in PBS for 1 h. Then, the medium was changed and cells were exposed to different *M. oleifera* leaf extract concentrations (10, 5, 3, and 1%). The control group was unexposed cells and cells treated with 1 mM hydrogen peroxide (H_2_O_2_) were used as a positive control. The DCF fluorescence was measured after 90 min of incubation, using a microplate reader FilterMax F5 (Thermo Fisher Scientific) at a maximum excitation of 485 nm and emission spectra of 530 nm.

To assay the capacity of model cosmetic formulation (1% SCS) containing various concentrations (5, 3, and 1%) of *M. oleifera* leaf extract to generate the intracellular level of reactive oxygen species, fluorogenic dye H2DCFDA was also used. HaCaT and BJ fibroblasts were seeded in 96-well plates at a density of 1 × 10^4^ cells per well and initially cultured for 24 h. After this, the culture medium was changed on 10 *µ*M H_2_DCFDA (Sigma) in serum-free medium (DMEM or MEM for HaCaT and fibroblasts, respectively). Cells were incubated with H_2_DCFDA for 45 min before treatment. After this, the medium was changed and cells were exposed to 1% SCS with different extract concentrations. The control group was untreated cells and cells treated with 1% of sodium coco sulfate (SCS) were used as a positive control. The DCF fluorescence was measured every 30 min for a total of 90 min using a microplate reader FilterMax F5 (Thermo Fisher Scientific) at a maximum excitation of 485 nm and emission spectra of 530 nm.

### 2.10. Statistical Analysis

Each value is the mean of three or five independent measurements. The values of the analyzed parameters were expressed as the mean ± standard deviation (SD). The two-way analysis of variance (ANOVA) and Bonferroni posttest between groups were performed at a *p* value of <0.05 to evaluate the significance of differences between values. Statistical analyses were performed using GraphPad Prism 5.0 (GraphPad Software, Inc., San Diego CA).

## 3. Results

### 3.1. Total Phenolic and Total Flavonoid Content Determination

**Figure 1:**
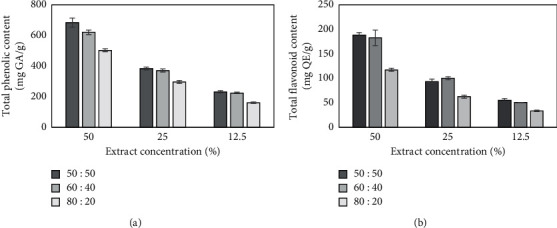
Total phenolic content (TPC) (a) and total flavonoid content (TFC) (b) in 50 : 50 (vol/vol), 60 : 40 (vol/vol), and 80 : 20 (vol/vol) aqueous/glycerin extract of *Moringa oleifera* leaves. Values are mean of three replicate determinations (*n* = 3) ± SD. The TPC and TFC amounts were calculated from the calibration curve (*R*^2^=0.9989 and *R*^2^=0.9996, respectively).

To confirm the previous result that *M. oleifera* is a rich source of antioxidant compounds, in this work an attempt was made to determine total phenolic content (TPC) and total flavonoid content (TFC). The amounts of these compounds were assayed from the calibration curves of gallic acid (*y*=0.0046*x*+0.0452, *R*^2^=0.9989) and quercetin (*y*=0.0153*x* − 0.0053, *R*^2^=0.9996), respectively. The analysis was performed for three different dilutions (50%, 25%, and 12.5%) of each extract. The obtained results show that the highest amount of phenols and flavonoids was characterized by 50 : 50 (vol/vol) aqueous/glycerin extract, while the lowest concentration of these compounds was characterized by 80 : 20 (vol/vol) aqueous/glycerin extract (Figure 1). The difference between the highest and the lowest TPC values for different types of 50% extract dilution was about 24%. Considering the TFC value, it was nearly 37%. It was also observed that the TPC and TFC were increasing in a dose-dependent manner for all tested types of *Moringa oleifera* leaf extracts.

### 3.2. Identification of Active Components

**Figure 2:**
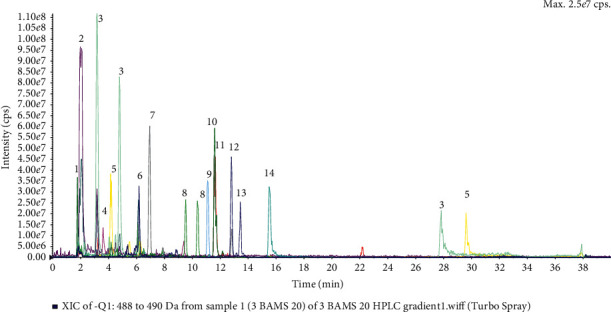
Extracted ion chromatogram (XIC) of the detected compounds in a representative sample.

The obtained results of HPLC-ESI-MS (Figure 2) in the negative ion mode revealed the presence of polyphenols, of which flavonoids and phenolic acids were the principal compounds. The observed flavonoids were quercetin and kaempferol derivatives, while phenolic acids were gallic, caffeic, quinic, and chlorogenic. The structures of the detected polyphenols were confirmed by MS^2^ fragmentation of selected *m*/*z* signals.  Table 1 lists polyphenols detected using ESI-MS/MS (ESI-MS analysis spectra are included as Supplementary material (available here)).

### 3.3. DPPH Radical Scavenging Assay

**Figure 3:**
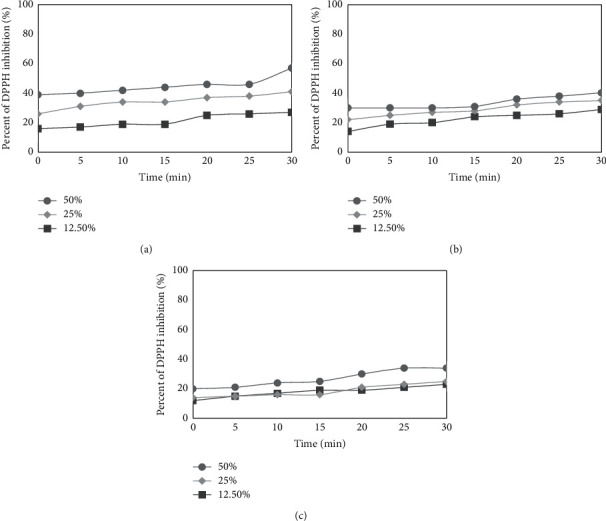
Kinetics of the absorbance changes in DPPH solutions in the presence of various concentrations of 50 : 50 (vol/vol) (a), 60 : 40 (b), and 80 : 20 (vol/vol) (c) aqueous/glycerin extract of *Moringa oleifera* leaves. Values are mean of three replicate determinations (*n* = 3).

To determine the antioxidant properties of all tested extracts, three different concentrations 12.5%, 25%, and 50% were used. The measurements were performed every five minutes over 30 min time period. The obtained data have shown that each concentration of the extract has a different ability to reduce free radicals (Figure 3). The highest ability to scavenge DPPH radical was shown by 50 : 50 (vol/vol) aqueous/glycerin extract at the highest tested concentration (50%). After 30 min of incubation, the level of reduced DPPH• was above 56%, while in the lowest concentration (12.5%), the level of scavenged radicals oscillated at 26%. Moringa tree 60 : 40 (vol/vol) aqueous/glycerin leaf extract was characterized by middling antioxidant ability in comparison with other extracts. The highest reducing power of this extract was observed for 50% of concentration and it was about 41% after 30 min of incubation. Considering the 80 : 20 (vol/vol) aqueous/glycerin extract, it has the lowest antioxidant potential. In the highest concentration, the ability to scavenge DPPH radical was only on 35% level. Furthermore, a relationship between used concentration and antioxidant potential of extracts was observed. When the concentration was increasing, the free radical reducing power was higher. The order of free radical scavenging capacity was as follows: 50 : 50 (vol/vol) > 60 : 40 (vol/vol) > 80 : 20 (vol/vol) aqueous/glycerin.

### 3.4. Cell Viability Assay

**Figure 4:**
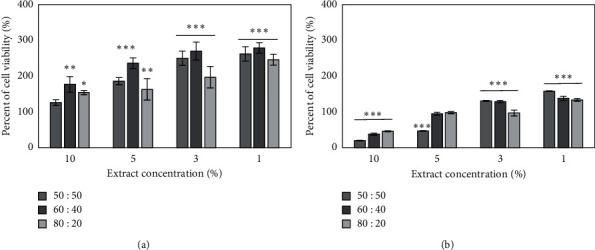
The effect of different concentrations of *Moringa oleifera* leaf extracts (10, 5, 3, and 1%) on resazurin salt reduction assay in cultured keratinocytes (a) and BJ fibroblast (b) after 24 h of exposure. Data are the mean ± SD of three independent experiments, each consisting of three replicates per treatment group. ^*∗∗∗*^*p* < 0.001, ^*∗∗*^*p* < 0.01, and ^*∗*^*p* < 0.05 versus the control.

The resazurin sodium salt reduction assay was used to assess cell viability. The obtained results indicate the cell-specific effect of the analyzed extracts on cell viability (Figure 4). In HaCaT cells, all tested concentrations as well as types of extracts showed a stimulating effect on cell viability. The highest difference between control and treated cells was observed for all types of 1% concentration of extracts. The best ability on increasing cell viability was shown for 60 : 40 (vol/vol) aqueous/glycerin extracts. It also has been observed that the cell proliferation was increasing in a dose-dependent manner. In turn, in BJ fibroblast, extracts showed both inhibitory and stimulatory effects. The highest concentrations (10% and 5%) of all tested extracts showed antiproliferative effect. For 10% of extract concentration, the fibroblast viability was observed on 20%, 37%, and 45% levels for 80 : 20 (v/v), 60 : 40 (v/v), and 50 : 50 (v/v), respectively, in comparison with the control. As well as, in HaCaT, the 1% of extract concentration has shown the most positive effect on cell viability. The highest response was observed for 50 : 50 (v/v) aqueous/glycerin extract and it was about 157%, in regard to the untreated group. The less cytotoxic potential was noted for 80 : 20 (v/v) *M. oleifera* leaf extract. It was also noticed that when the extract concentration was decreased, the cell viability increased.

### 3.5. Measurement of DCF Fluorescence

**Figure 5:**
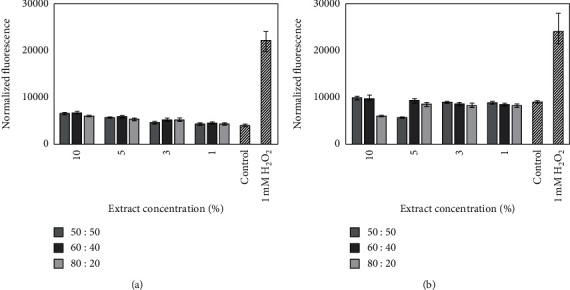
The effect of various concentrations of *Moringa oleifera* leaf extracts (10, 5, 3, and 1%) on the DCF fluorescence in keratinocytes (a) and BJ fibroblast (b) cells preincubated with hydrogen peroxide (H_2_O_2_). Medium with 1 mM hydrogen peroxide (H_2_O_2_) was used as a positive control. The data are expressed as the mean ± SD of three independent experiments, each consisting of three replicates per treatment group.

After preincubation of cells with 1 mM H_2_O_2_, the intracellular level of produced reactive oxygen species level robustly increased in both HaCaT and BJ, compared to the nontreated cells. After 90 min of cell treatment with various concentrations of all types of extracts, the level of ROS significantly decreased to the level oscillating to the control group, which were untreated cells (Figure 5).

### 3.6. Cell Viability Assay and Measurement of DCF Fluorescence of Model Formulation of Cosmetic

**Figure 6:**
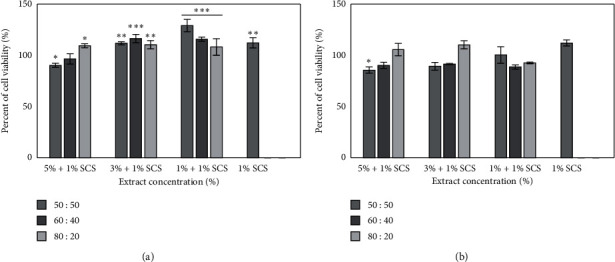
The effect of different model base formulations of cosmetic (1% SCS) containing various concentrations of *Moringa oleifera* leaf extracts (5, 3, and 1%) on resazurin reduction in cultured keratinocytes (a) and fibroblast (b) after 30 min of exposure. Data are the mean ± SD of three independent experiments, each consisting of three replicates per treatment group. ^*∗∗∗*^*p* < 0.001, ^*∗∗*^*p* < 0.01, and ^*∗*^*p* < 0.05 versus the control.

**Figure 7:**
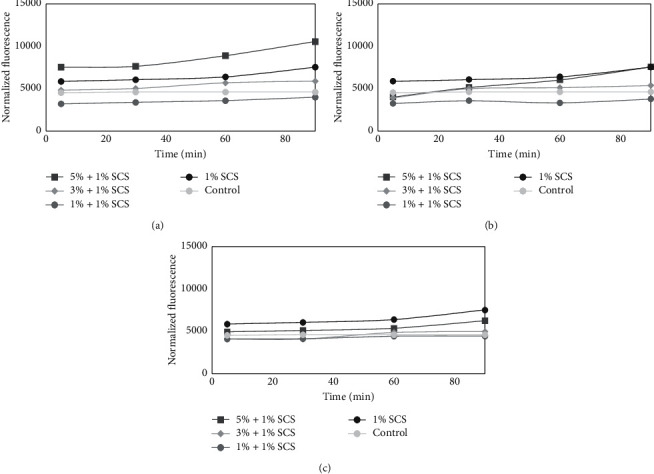
The effect of different model base formulation of cosmetic (1% SCS) containing various concentrations of 50 : 50 (vol/vol) (a), 60 : 40 (b), and 80 : 20 (vol/vol) (c) *Moringa oleifera* leaf extracts on the DCF fluorescence in normal human keratinocyte cells. Unexposed cells were used as a control. The data are expressed as the mean ± SD of three independent experiments, each consisting of three replicates per treatment group.

**Figure 8:**
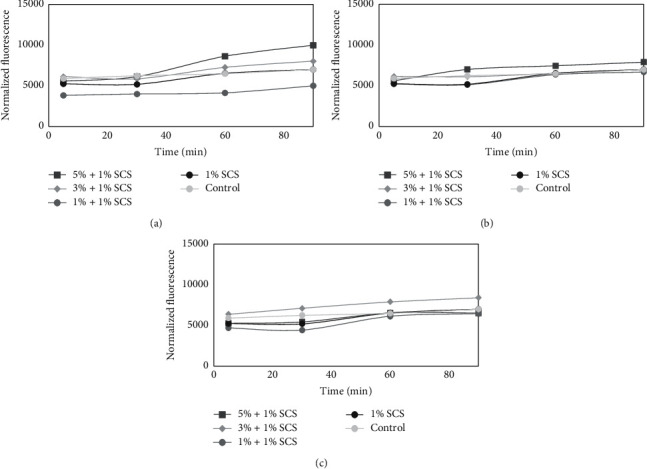
The effect of different model base formulation of cosmetic (1% SCS) containing various concentrations of 50 : 50 (vol/vol) (a), 60 : 40 (b), and 80 : 20 (vol/vol) (c) *Moringa oleifera* leaf extracts on the DCF fluorescence in fibroblast cells. Medium with 1% of sodium coco sulfate (SCS) was used as a positive control. The data are expressed as the mean ± SD of three independent experiments, each consisting of three replicates per treatment group.

To evaluate if model cosmetic formulation (1% SCS) containing various concentrations (10, 5, 3, and 1%) of *Moringa oleifera* leaf extract has an effect on cell viability, the resazurin sodium salt reduction assay was used. Obtained data indicated that in HaCaT cells, tested extracts did not induce any significant decrease in cell viability in comparison with the control cells. Furthermore, it was noticed that base (1% SCS) plus 1% or 3% of extracts can significantly increase cell metabolism. The highest increase in cell viability was observed for 1% SCS + 1% of 50 : 50 (vol/vol) aqueous/glycerin extract and it was about 30% higher than in the control group. It can be also noted that base (1% SCS) did not induce toxic effect on HaCaT cells. In turn, in BJ fibroblast cells, the substances tested exhibited the opposite effect. No toxic or proliferative effect on cell viability was observed for all types of extracts and concentrations tested. Slight increase of BJ cell metabolism was noticed for 1% SCS + 5% and 1% SCS + 3% of 80 : 20 (vol/vol) aqueous/glycerin extract (104 and 106%, respectively). Percent of viable cells after treatment with 1% of SCS was little under the control group (Figure 6). To assay the capacity of model cosmetic formulation (1% SCS) containing various concentrations (5, 3, and 1%) of *M. oleifera* leaf extract to generate intracellular level of reactive oxygen species, fluorogenic dye H2DCFDA was also used. The reactive oxygen species production was measured by the use of H_2_DCFDA method. As the results showed, the 60 : 40 (v/v) and 80 : 20 (v/v) aqueous/glycerin Moringa tree leaf extract did not significantly generate oxidative stress in HaCaT cells. The highest ROS production was exhibited by 1% SCS. In addition, the most intracellular increase in ROS level was noticed for 1% SCS + 5% 50 : 50 (v/v) extract and it was twofold higher than that of the nontreated group. Values for other types of extracts and concentrations oscillate about the control. It was also observed that when the extract concentration decreased, the amount of ROS in cells was also lower (Figure 7). Considering the results of BJ fibroblast, it has been shown that the highest intracellular level of reactive oxygen production was for 1% SCS + 5% of 50 : 50 (v/v) extract compared to the untreated group. Values for other concentrations of this extract were similar to the unexposed cells. For 60 : 40 (v/v) and 80 : 20 (v/v) aqueous/glycerin *M. oleifera* leaf extract, no changes in the induction of oxidative stress were observed. The level of intracellular ROS oscillated around the control group (Figure 8).

### 3.7. Zein Test

**Figure 9:**
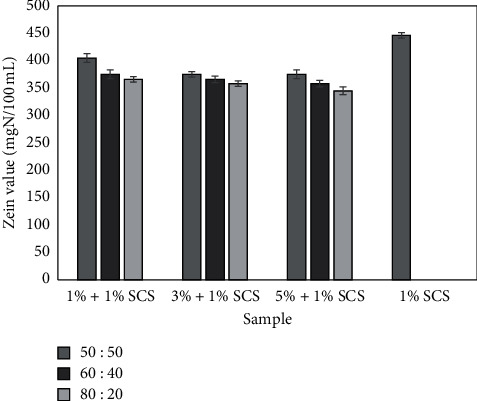
Irritant potential of model products containing 1.0 wt% SCS and extract from *Moringa oleifera L.* (1%, 3%, and 5%).

To assess the irritant potential of the products, the zein test was used. The results showed that the *M. oleifera* leaf extracts might improve the safety of use of the model washing system in terms of its effect on the skin. The addition of the analyzed extracts to 1% SCS causes a decrease in the value of the zein number and thus reduces skin irritation potential. The value of the measured parameter in the base sample was at 451 mgN/100 mL. The test results are presented in Figure 9. The skin irritation potential of the samples with the addition of extracts was about 10–20% lower than the baseline. It was observed that the extract concentrations used did not significantly affect the value of zein number, and the differences noted between the samples were within the margin of error. No significant influence of the extractant on the analyzed parameter was observed.

## 4. Discussion

The major group of phytochemicals contributing to the antioxidant capacity of plant material includes polyphenols, carotenoids, and vitamins C and E. There are several previous studies, which show that *M. oleifera* leaves are rich source of bioactive compounds with antioxidant properties. It has been reported that Moringa tree leaves have sufficient amounts of quercetin, kaempferol, *β*-carotene, *α*-tocopherol, and vitamin A [36, 37].

With regard to these findings, the present investigation was undertaken to evaluate the antioxidant capacity of aqueous/glycerin extracts of *M. oleifera* leaves. We were shown that leaf extract exhibited a high content of phenols and flavonoids. The obtained results show that the highest amount of phenols and flavonoids was found in 50 : 50 (vol/vol) aqueous/glycerin extract, while the lowest concentration of these compounds was found in 80 : 20 (vol/vol) aqueous/glycerin extract. As it was previously mentioned, the most important properties of phenolic compounds derived from plant material are their antioxidant properties. The phenols consist of a hydroxyl group and play an essential role in the antioxidant ability by donating hydrogen and forming stable radical intermediates [38]. The mechanism of action of phenols mainly relies on neutralization of free radicals, chelation of metal ions, and induction of dismutase enzymes, as well as peroxidases [39].

The phytochemical profiles of *Moringa oleifera* were studied by ESI-MS. Via the screening analysis, the presence of polyphenols in investigated *Moringa oleifera* extracts was identified of which phenolic acids and flavonoids were the principal compounds (Table 1). Phenolic acids and derivatives such as gallic acid [M-H]^−^ at *m/z* 169, caffeic acid [M-H]^−^ at *m/z* 179, quinic acid [M-H]^−^ at *m/z* 191, p-coumaroylquinic acid [M-H]^−^ at *m/z* 337, and chlorogenic acid [M-H]^−^ at *m/z* 353 were detected according to ESI-MS analysis, and the structures were confirmed by MS^2^ fragmentation. Flavonols were found in the studied samples being quercetin derivatives (MS^2^ fragment at *m/z* 300 and 270–272). Quercetin [M-H]^−^ at *m/z* 301, isoquercetin [M-H]^−^ at *m/z* 463, quercetin-acetyl-glucoside [M-H]^−^ at *m/z* 505, quercetin-malonylhexoside [M-H]^−^ at *m/z* 549, and rutin [M-H]^−^ at *m/z* 609 were found in the studied extracts, and the structures were confirmed by MS^2^ fragmentation. Kaempferol derivatives (MS^2^ fragment at *m/z* 284) found in the extracts were kaempferol-3-O-glucoside [M-H]^−^ at *m/z* 447, kaempferol-acetyl-glucoside [M-H]^−^ at *m/z* 489, and kaempferol-3-O-rutinoside [M-H]^−^ at *m/z* 593.

Phytochemical analysis with ESI-MS was conducted to correlate the antioxidant activities with chemical compositions of *Moringa oleifera*. The results indicated that leaves of *Moringa oleifera* are rich in phenolic compounds and thus have a potential to be used as source of components with antioxidant activities. The high amounts of phenolic compounds in *Moringa oleifera* as a potential source of phytochemicals were also pointed out by Özcan et al. [20].

The next stage of this work was to evaluate the ability of *M. oleifera* leaf extracts to scavenge free radicals. For this, we used DPPH• reducing assay, where changes of color solution are directly linked with the decrease of absorption values and the number of formed DPPH radicals [40]. Results from DPPH• scavenging assay obtained in this paper correspond to the other research. Atawodi et al. [36] in their work indicated on strong antioxidant activity of ethanolic extracts from different parts *M. oleifera* ethanolic extracts. Using the 2-deoxyguanosine assay model, he has shown that the highest free radical scavenging activity was characterized by leaves. Remarkable antioxidant activity of drumstick leaves might be possessed due to the presence of quercetin and kaempferol as well as chlorogenic acids and their derivatives, which were detected in this part of the plant [36]. In addition, it was hypothesized that antioxidant activity of *M. oleifera* leaf extracts could be accomplished through donating protons as well as reductones, which exert activity by breaking the free radical chain [41]. The biological activity of leaves of *Moringa oleifera* was also investigated on cells as *in vitro* model. The cytotoxicity of tested extracts was assessed on HaCaT and BJ fibroblast cell lines using resazurin method. Both cell types were treated with various extract concentrations, ranging from 1% up to 10% in the cultured medium. The obtained results indicate the cell-specific and dose-dependent effect of the analyzed extracts on cell viability. Whereas in HaCaT cells all tested concentrations as well as types of extracts showed stimulating effect on cell viability, in fibroblasts extracts showed both inhibitory and stimulatory effects. The equal connection in cell viability in response to increasing concentration of *M. oleifera* leaf extracts was also observed by other researchers. The antiproliferative effect was shown in KB (tumor cells), HEK-293 (human lung carcinoma), and A549 cells (human embryonic kidney cells). These papers indicated that antiproliferative effect was associated with induction of apoptosis, morphological changes, and DNA fragmentation [37, 42, 43]. The mechanism of cell death may be reflected in the above reports; however, to be confirmed, a more thorough analysis should be carried out. In the next stage of this experiment, it has been evaluated if *M. oleifera* leaf extracts could inhibit H_2_O_2_-induced ROS production on *in vitro* cell models. As a substrate to determine the intracellular formation of ROS generation, H_2_DCFDA assay was used. As is recommended, we examined whether plant extracts without cells affected the fluorescence of the H_2_DCFDA [44]. Additionally, the separated experiment showed that there were no interactions between plant extracts and H_2_DCFDA substrate in DMEM or MEM. This experiment has shown that *M. oleifera* leaf extract possess antioxidant properties. The tested extracts were able to significantly decrease the high number of intracellular ROS to the level oscillating to the control group, which were untreated cells. The high ability of extracts to reduce oxidative stress might be correlated with rich content of phenols and flavonoids. According to Vongsak et al., it was directly indicated that major bioactive substances present in leaf extracts that provide strong inhibition of H_2_O_2_-induced oxidative stress are cryptochlorogenic acid and isoquercetin [42]. Given the antioxidant nature of tested substances, based on the previous experience and available information, a model washing gel formula containing *M. oleifera* leaf extract was prepared. Resazurin reduction assay has been used to check if model cosmetic formulation (1% sodium coco sulfate—SCS) with various concentrations (5, 3, and 1%) of tested extracts has an effect on cell viability. The obtained results indicated that the use of the extract in the cosmetic formula is not dangerous to human skin cells and additionally introduces the desired properties such as antioxidant activity. In order to further confirm the above reports, the ability of model cosmetic formulation containing *M. oleifera* leaf extract to generate intracellular ROS in keratinocytes and fibroblast was assayed. The reactive oxygen species production was measured using the H_2_DCFDA method. The above findings correlate with the cell viability assay, and therefore, it seems that all types of tested extracts are a desirable plant material with remarkable antioxidant activity. These features can protect it from aging and other diseases, where the reactive oxygen plays major role. Moringa tree leaf extracts were also tested by evaluating its ability to reduce the irritant potential of sodium coco sulfate (SCS), the anionic surfactant used in the formulation of body wash cosmetics. For this purpose, four samples constituting model washing systems were prepared. Each sample contained 1 wt% of SCS combined with 1, 3, and 5 wt% of the extracts prepared with different ratios of water and glycerin as extractant. The reference (baseline) sample contained 1 wt% solution of SCS without any extract. The pH of each sample was adjusted with 25% citric acid to a value of 5.5, the physiological pH value of the skin. The irritant potential of model body wash systems was analyzed by zein value measurements. In the surfactants, solution zein protein is denatured and then is solubilized in the solution. This process simulates the behavior of surfactants in relation to the skin proteins. According to the literature [2–4, 7, 8, 10–13, 45, 46], the sample should be classified as highly irritating for the value of the zein number exceeding 400 mgN/100 mL. The skin irritation potential of the samples with the addition of extracts was about 10–20% lower than the baseline. It was observed that the extract concentrations used did not affect the value of zein number, and the differences noted between the samples were within the margin of error. The decrease in the zein number following the application of the extract to the model cosmetic formula might be the result of the presence of active substances in the extracts. In aqueous solutions and at low concentrations, surfactants occur in the form of individual particles referred as monomers [2, 4, 5, 10–13]. When specific concentration unique to a compound is reached, referred as the critical micelle concentration (CMC), the micellar aggregates start to appear in surfactant solutions. The irritation potential is significantly correlated with the type and concentration of the surface active agent. The highest skin irritation ability is attributed to anionic surfactants and surfactants present in the form of monomers. They demonstrate the capacity to form strong and long-lasting bonds with epidermal proteins. Following their ability to bind with proteins, surfactants may cause denaturation and elimination (elution) proteins from the skin, which results as a cutaneous irritation. Available data indicate that the irritant potential might be reduced by bindings of monomers with various types of substances including peptides, polymers, polysaccharides, and mineral salts, where all of them were found in plant extracts [2, 4, 5, 10–13, 45–52]. Another factor which is potentially contributing to a reduction of irritant potential is the stabilization of micelles formed in solutions. Micelles are thermodynamically unstable aggregates, which constantly disintegrates and releases monomers into the volume phase of the solution. Stabilization of micelles in the presence of plant extracts may take place through the incorporation of such substances as proteins, polyphenols, and flavonoids, as well as solvents used in the extraction process (glycerin and glycols) into their structure [2, 4, 5, 10–13, 46–53]. The effect of Moringa extracts on the irritation potential of surfactants has not been studied before. Our previous research indicates that plant extracts may reduce the irritation potential of cleansing cosmetics and surfactants. It has been shown that the addition of dogwood extracts [13], natural saponins [11], blackcurrant [8], or chicory [54] reduces the irritation potential of cleansing cosmetics and anionic surfactants.

## 5. Conclusions

In the present paper, an attempt was made to determine the properties and the applicability of extracts from *Moringa oleifera* leaves in model products. The tested extracts were characterized by a high content of phenolic compounds, flavonoids, and high antioxidant potential. *In vitro* toxicity studies showed that the tested extracts in concentrations up to 5% showed a positive effect on cell proliferation and metabolism. It has also been shown that the extracts may contribute to the reduction of oxidative stress in cells. It was noted that the tested model formulation of cosmetic (1% SCS) with the addition of different types of extracts in various concentrations does not negatively affect cell metabolism. Analyses defining the ROS level showed that model cosmetic formulation (1% SCS) with the presence of tested extracts does not cause an increase in the formation of intracellular reactive forms of oxygen. To summarize, the results of the research conducted show that application of extracts from the *Moringa oleifera* leaves to the model cosmetic formulation might contribute to the reduction of skin irritation and improve the safety of the product.

## Figures and Tables

**Table 1:** Polyphenols detected using ESI-MS/MS.

No.	Retention time (min)	Molecular formula	Molar mass (Da)	Precursor ion [M-H]^−^*m*/*z*	Main productions MS^2^*m*/*z*	Identification
1	2.0	C_7_H_12_O_6_	192.2	191	171 [M-H-H-H-H_2_O]^−^,127, [M-H-H_2_O-HCOOH]^−^, 85 [M-C_3_H_7_O_4_]^−^	Quinic acid
2	2.1	C_9_H_8_O_4_	180.2	179	135 [M-COOH]^−^, 107 [M-C_3_H_5_O_2_]^−^, 71 [M-C_6_H_5_O_2_]^−^, 59 [M-C_7_H_5_O_2_]^−^	Caffeic acid
3	3.2; 4.8; 28.0	C_16_H_18_O_9_	354.3	353	191 [M-3H_2_O-C_6_H_5_O_2_]^−^, 179 [M-3H_2_O-C_6_H_4_-COOH]^−^, 135 [M-3H_2_O-C_6_H_4_-C_2_HO_4_]^−^	Chlorogenic acid
4	3.6	C_7_H_6_O_5_	170.1	169	151 [M-H-H_2_O]^−^, 125 [M-H-CO_2_]^−^, 107 [M-H-CO_2_-H_2_O]^−^, 83 [M-C_3_H_3_O_3_]^−^	Gallic acid
5	4.1; 29.6	C_16_H_18_O_8_	339.0	337	191 [M-C_9_H_7_O_2_]^−^, 173 [M-H-C_9_H_7_O_3_]^−^, 163 [M-3 H_2_O-C_7_H_5_O_2_]^−^, 119 [M-C_8_H_11_O_7_]	Coumaroylquinic acid
6	6.2; 12.8	C_21_H_20_O_11_	448.3	447	284 [M-H_2_O-C_6_H_10_O_4_]^−^, 255 [M-C_6_H_9_O_7_]^−^, 179 [M-C_15_H_9_O_5_]^−^	Kaempferol-3-O-glucoside
7	6.9	C_27_H_30_O_15_	594.5	593	383 [M-C_8_H_19_O_6_]^−^, 352 [M-C_9_H_21_O_7_]^−^, 284 [M-C_12_H_22_O_9_]^−^	Kaempferol-3-O-rutinoside
8	9.5; 10.4	C_16_H_18_O_9_	432.1	431	340 [M-H-C_3_H_7_O_3_]^−^, 311 [M-C_4_H_9_O_4_]^−^, 179 [M-C_15_H_9_O_4_]^−^	Apigenin-glucoside
9	11.1	C_27_H_30_O_16_	610.5	609	300 [M-H-C_12_H_21_O_9_]^−^, 270 [M-H-C_12_H_21_O_9_-CHO]^−^	Rutin
10	11.6	C_23_H_22_O_13_	506.4	505	300 [M-H_2_O-C_6_H_8_O_4_-COCH_3_]^−^, 271 [M-H_2_O-C_6_H_9_O_4_-COCH_3_-CHO]^−^, 255 [M-C_6_H_15_O_8_], 179 [M-C_15_H_7_O_6_-COCH_3_]^−^	Quercetin-acetyl-glucoside
11	11.6	C_24_H_22_O_15_	550.4	549	505 [M-CH_2_O]^−^, 300 [M-C_9_H_14_O_8_]^−^, 463 [M-C_3_H_3_O_3_]^−^	Quercetin-malonylhexoside
12	12.8	C_21_H_20_O_12_	464.1	463	300 [M-H_2_O-C_6_H_10_O_4_]^−^, 271 [M-H_2_O-C_6_H_10_O_4_-CHO]^−^, 179 [M-C_15_H_9_O_6_]^−^	Isoquercetin
13	13.4	C_23_H_22_O_12_	490.4	489	284 [M-H_2_O-C_6_H_9_O_4_-COCH_3_]^−^, 254 [M-H_2_O-C_6_H_10_O_4_-COCH_3_-CHO]^−^, 178 [M-H-C_15_H_8_O_5_-COCH_3_]^−^	Kaempferol-acetyl-glucoside
14	15.5	C_15_H_10_O_7_	302.2	301	272 [M-CHO]^−^, 255 [M-H-CO-H_2_O]^−^, 151 [M-C_8_H_7_O_3_]^−^, 121 [M-C_8_H_5_O_5_]^−^, 107 [M-C_9_H_5_O_5_]^−^	Quercetin

## Data Availability

Readers can access the data underlying the findings of this study by contacting the author through tbujak@wsiz.rzeszow.pl.

## Supplementary Materials

Supplementary Materials Supplementary material contains spectra from composition analyses using ESI-MS. Figure 1: ESI-MS spectrum in the negative ion mode obtained for *Moringa oleifera* extract. Figure 2: ESI-MS spectrum in the negative ion mode obtained for *Moringa oleifera* extract, *m*/*z* 100–250 Da. Figure 3: ESI-MS spectrum in the negative ion mode obtained for *Moringa oleifera* extract, 80 : 20. Figure 4: ESI-MS spectrum in the negative ion mode obtained for *Moringa oleifera* extract, 60 : 40. Figure 5: ESI-MS spectrum in the negative ion mode obtained for *Moringa oleifera* extract, 50 : 50. Figure 6: MS^2^ fragmentation of gallic acid. Figure 7: MS^2^ fragmentation of caffeic acid. Figure 8: MS^2^ fragmentation of quinic acid. Figure 9: MS^2^ fragmentation of quercetin. Figure 10: MS^2^ fragmentation of p-coumaroylquinic acid. Figure 11: MS^2^ fragmentation of chlorogenic acid. Figure 12: MS^2^ fragmentation of apigenin-glucoside. Figure 13: MS^2^ fragmentation of kaempferol-3-O-glucoside. Figure 14: MS^2^ fragmentation of isoquercetin. Figure 15: MS^2^ fragmentation of kaempferol-acetyl-glucoside. Figure 16: MS^2^ fragmentation of quercetin-acetyl-glucoside. Figure 17: MS^2^ fragmentation of quercetin-3-O-malonylhexoside. Figure 18: MS^2^ fragmentation of kaempferol-3-rutinoside. Figure 19: MS^2^ fragmentation of rutin.
