# Supplementary material for: Moringa oleifera L. Extracts as Bioactive Ingredients That Increase Safety of Body Wash Cosmetics
Source: Dermatol Res Pract. 2020 Jul 1;2020:8197902. doi: 10.1155/2020/8197902 (PMC7350073; doi:10.1155/2020/8197902)
Supplement: Supplementary Materials — Supplementary material contains spectra from composition analyses using ESI-MS. Figure 1: ESI-MS spectrum in the negative ion mode obtained for Moringa oleifera extract. Figure 2: ESI-MS spectrum in the negative ion mode obtained for Moringa oleifera extract, m/z 100–250 Da. Figure 3: ESI-MS spectrum in the negative ion mode obtained for Moringa oleifera extract, 80 : 20. Figure 4: ESI-MS spectrum in the negative ion mode obtained for Moringa oleifera extract, 60 : 40. Figure 5: ESI-MS spectrum in the negative ion mode obtained for Moringa oleifera extract, 50 : 50. Figure 6: MS2 fragmentation of gallic acid. Figure 7: MS2 fragmentation of caffeic acid. Figure 8: MS2 fragmentation of quinic acid. Figure 9: MS2 fragmentation of quercetin. Figure 10: MS2 fragmentation of p-coumaroylquinic acid. Figure 11: MS2 fragmentation of chlorogenic acid. Figure 12: MS2 fragmentation of apigenin-glucoside. Figure 13: MS2 fragmentation of kaempferol-3-O-glucoside. Figure 14: MS2 fragmentation of isoquercetin. Figure 15: MS2 fragmentation of kaempferol-acetyl-glucoside. Figure 16: MS2 fragmentation of quercetin-acetyl-glucoside. Figure 17: MS2 fragmentation of quercetin-3-O-malonylhexoside. Figure 18: MS2 fragmentation of kaempferol-3-rutinoside. Figure 19: MS2 fragmentation of rutin. [file 8197902.f1.pdf]

Moringa oleifera L. extracts as bioactive ingredients that increasing safety of body wash cosmetics

Zofia Nizioł-Łukaszewska, Dominika Furman-Toczek, Tomasz Bujak, Tomasz Wasilewski, Zofia Hordyjewicz-Baran

Supplementary material contains spectra from composition analyzes using ESI-MS.

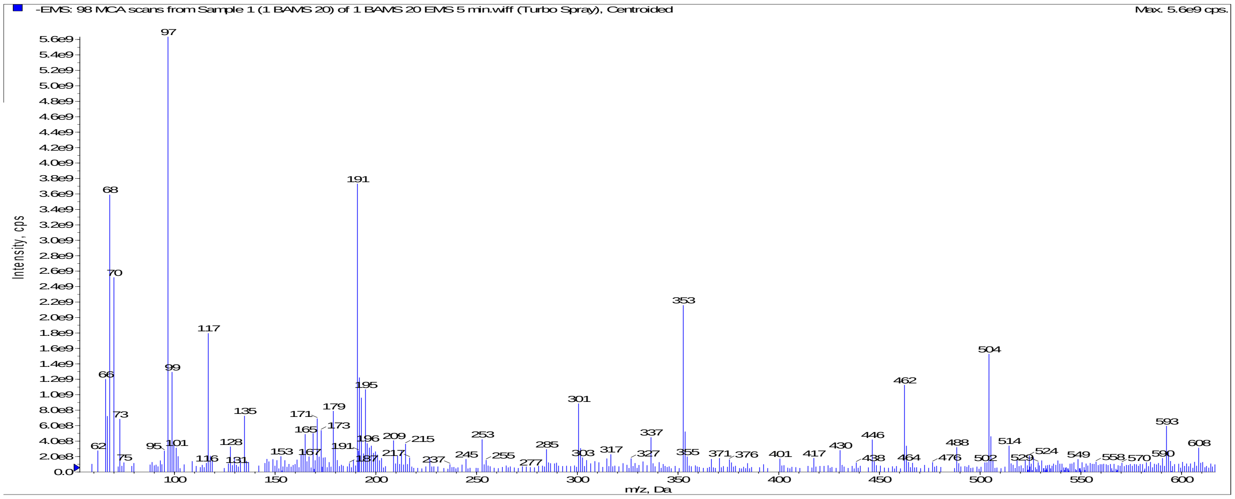

Figure 1. ESI-MS spectrum in the negative-ion mode obtained for *Moringa oleifera* extract.

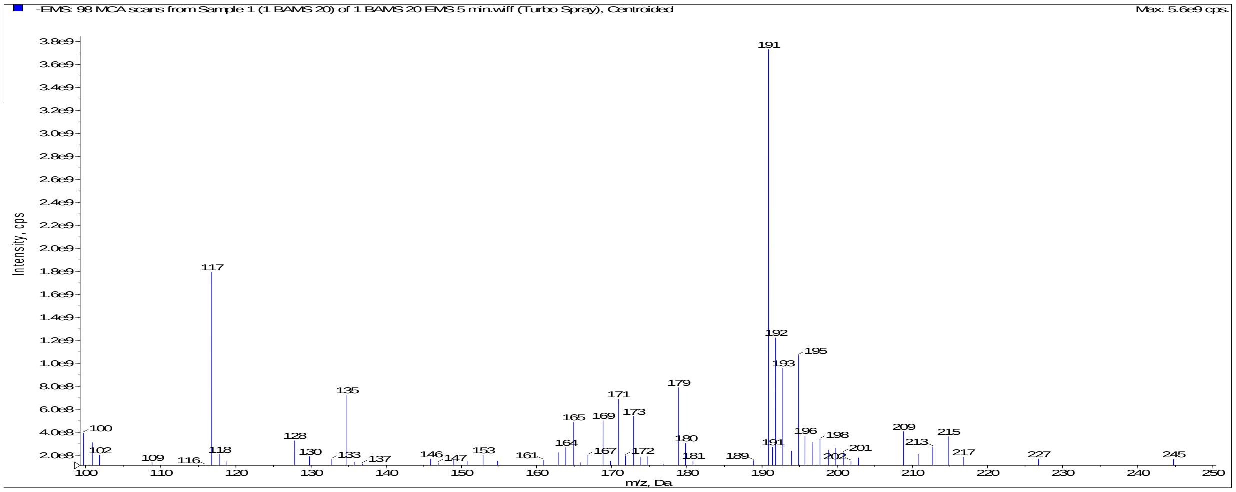

Figure 2. ESI-MS spectrum in the negative-ion mode obtained for *Moringa oleifera* extract, m/z 100-250 Da.

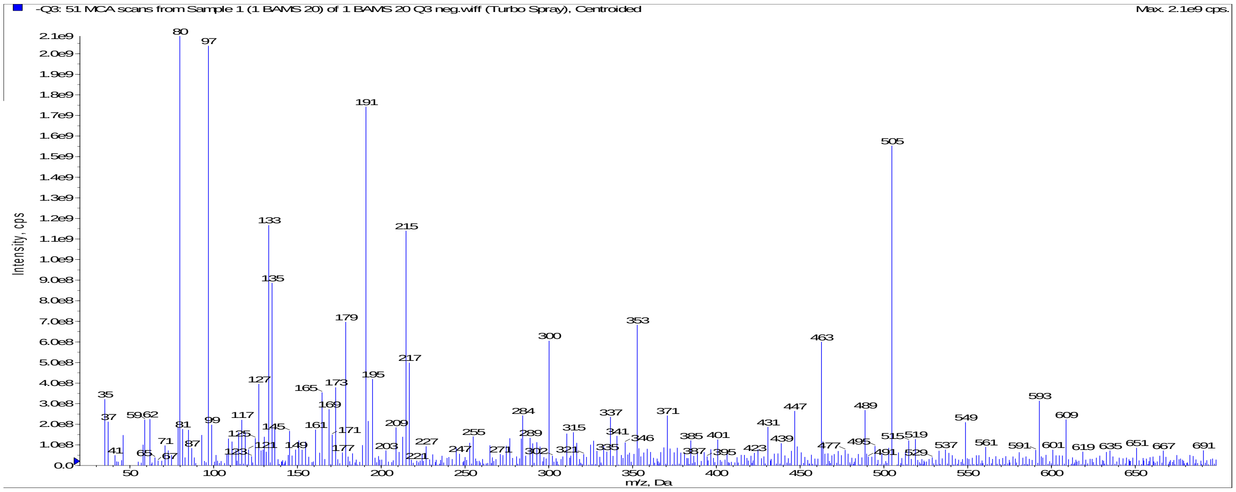

Figure 3. ESI-MS spectrum in the negative-ion mode obtained for *Moringa oleifera* extract, 80:20.

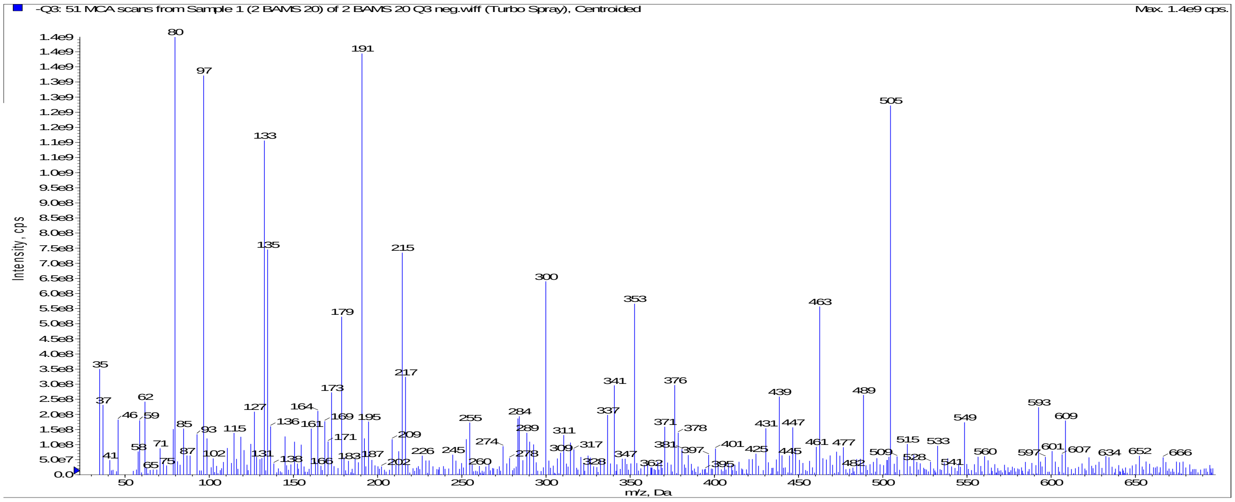

Figure 4. ESI-MS spectrum in the negative-ion mode obtained for *Moringa oleifera* extract, 60:40.

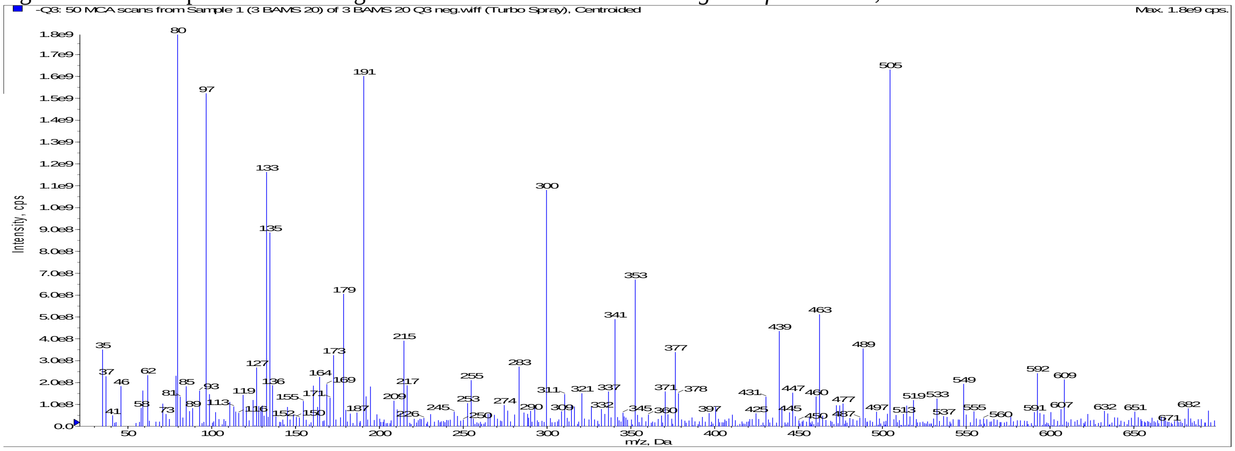

Figure 5. ESI-MS spectrum in the negative-ion mode obtained for *Moringa oleifera* extract, 50:50.

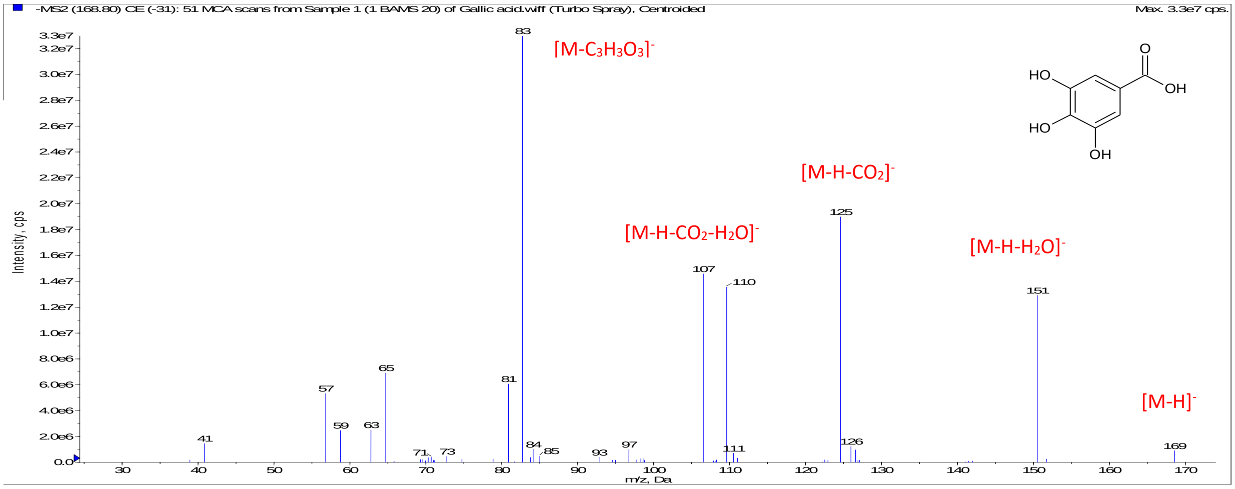

Figure 6. MS[2] fragmentation of Gallic acid.

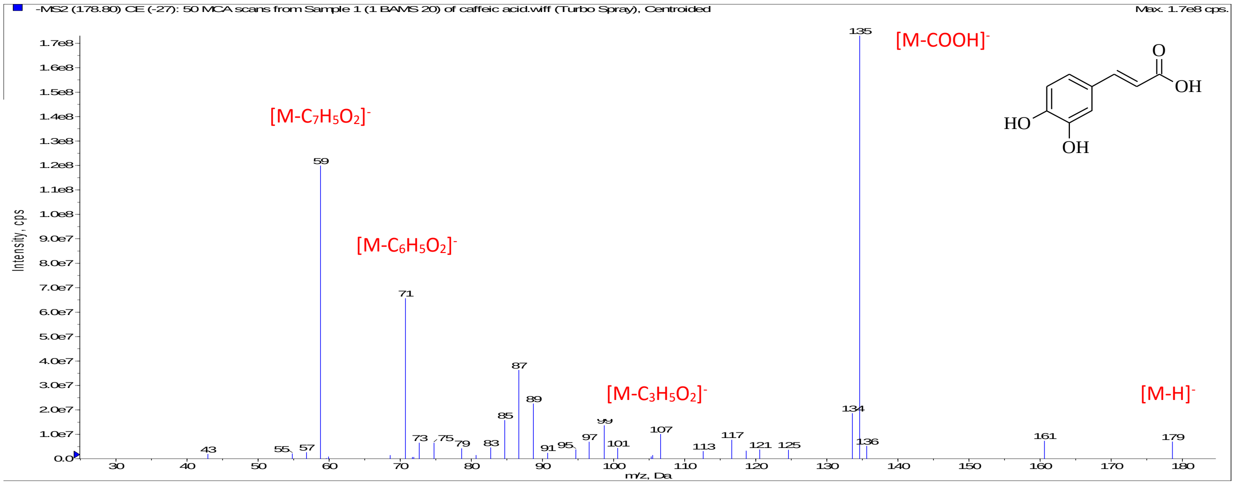

Figure 7. MS[2] fragmentation of Caffeic acid.

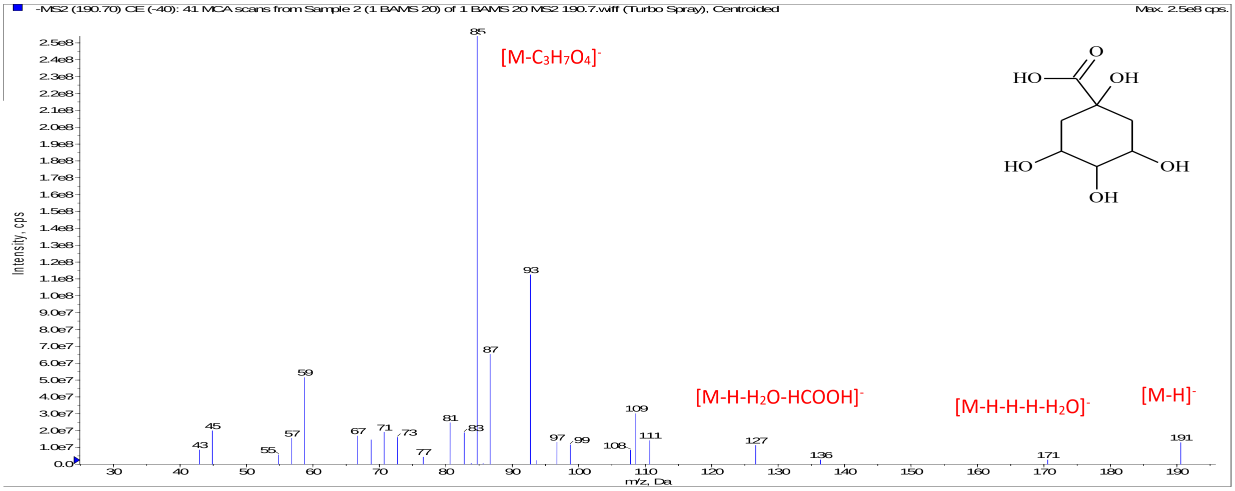

Figure 8. MS[2] fragmentation of Quinic acid .

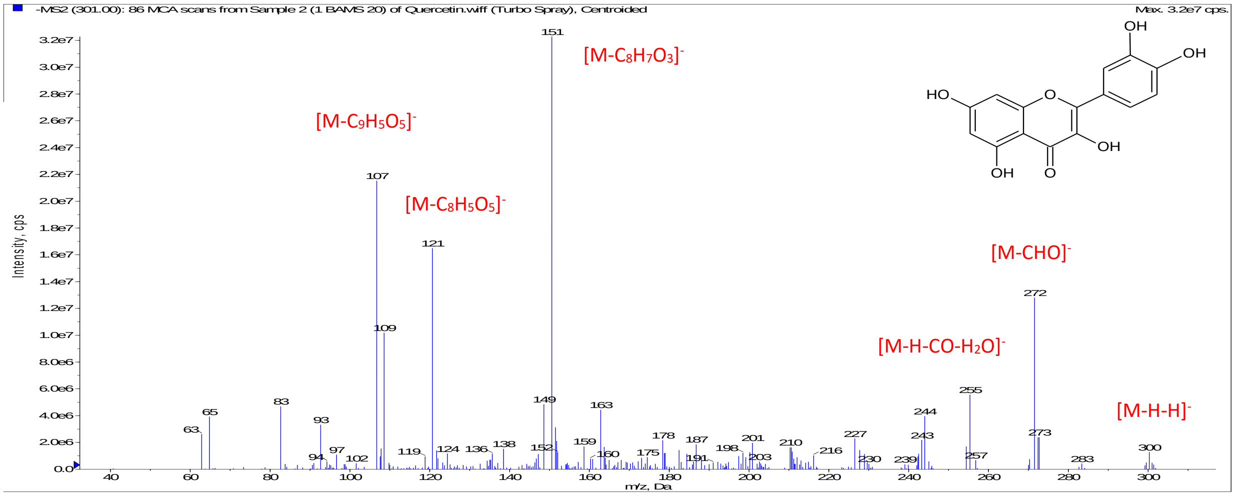

Figure 9. MS^2 fragmentation of Quercetin.

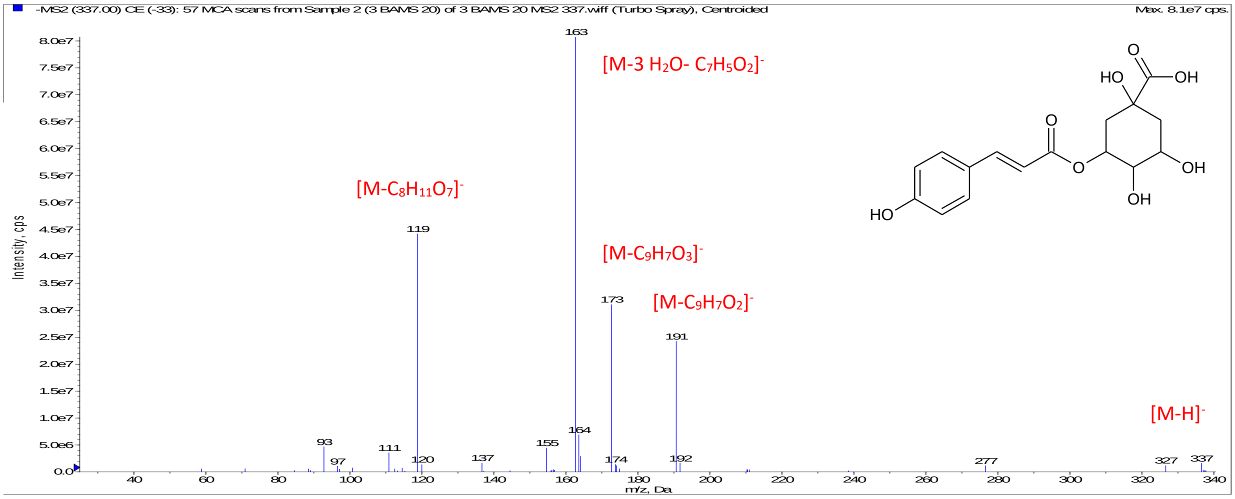

Figure 10. MS^2 fragmentation of p-Coumaroylquinic acid.

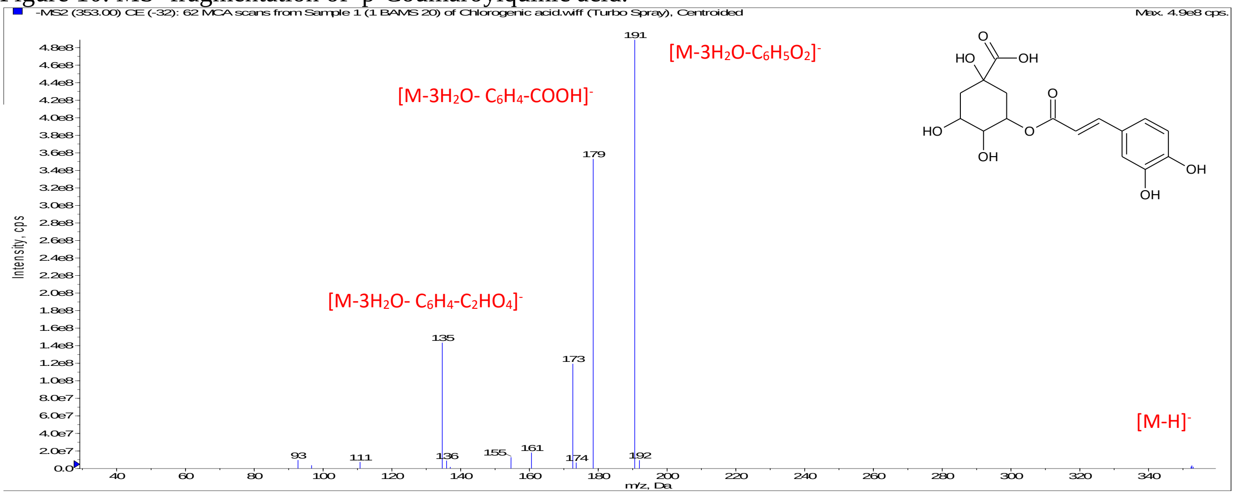

Figure 11. MS^2 fragmentation of Chlorogenic acid.

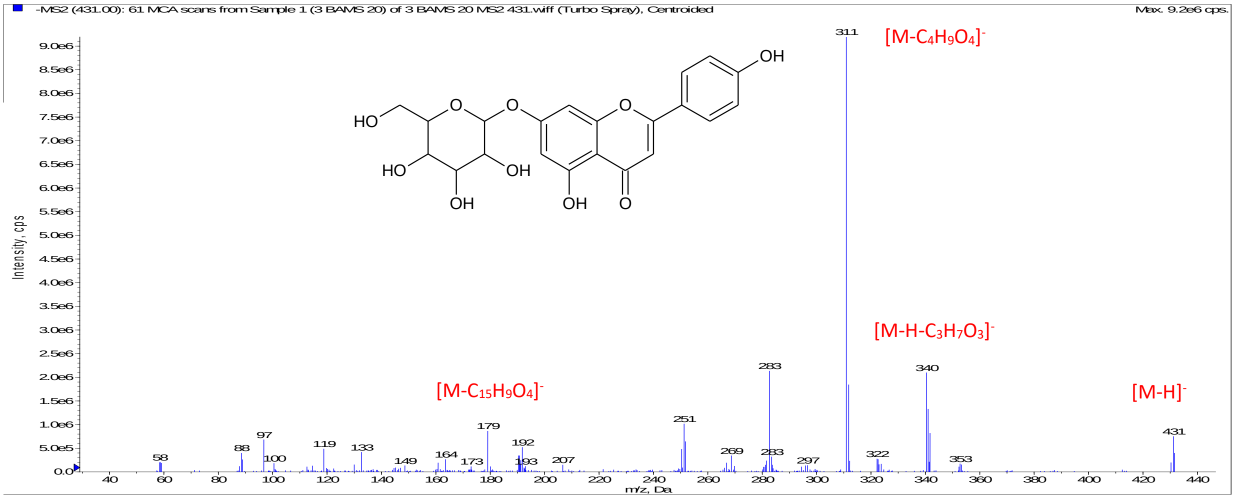

Figure 12. MS^2 fragmentation of Apigenin-glucoside.

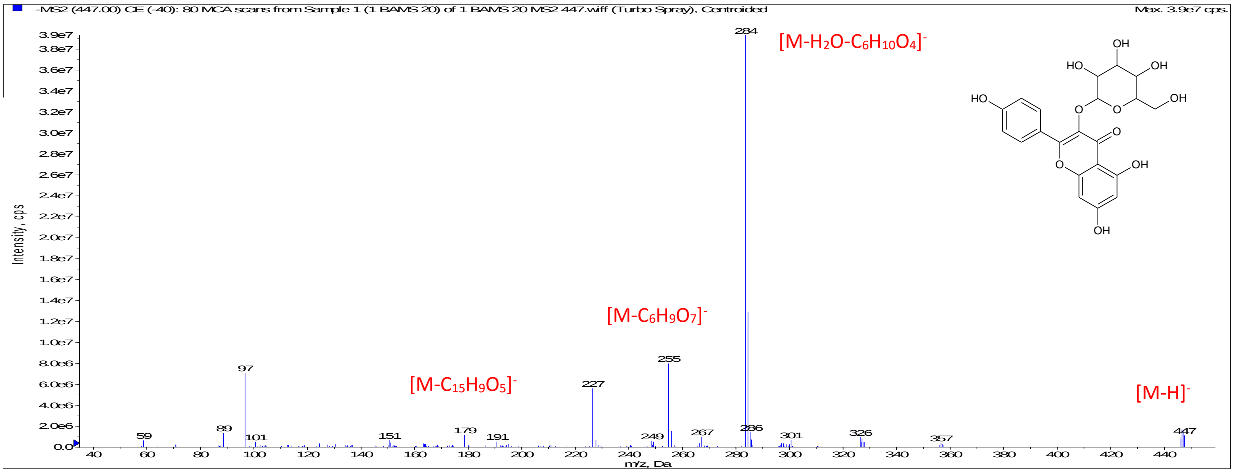

Figure 13. MS^2 fragmentation of Kaempferol -3-O-glucoside.

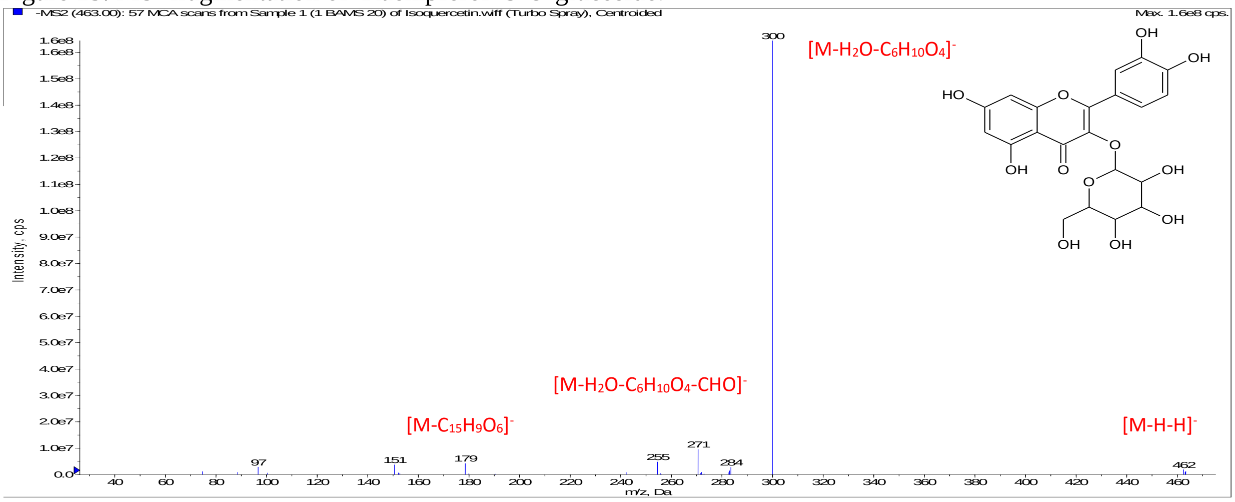

Figure 14. MS^2 fragmentation of Isoquercetin.

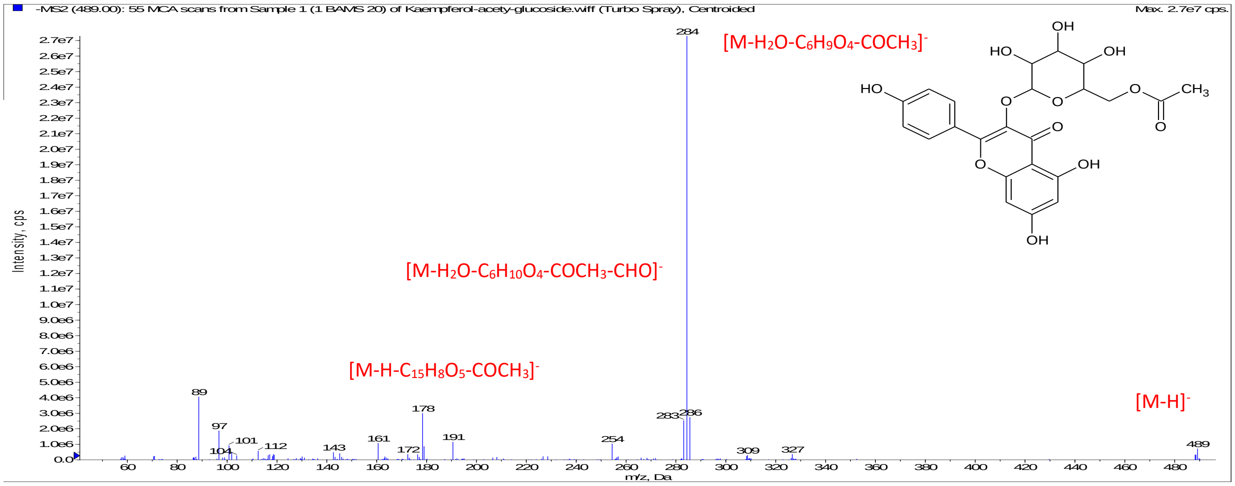

Figure 15. MS^2 fragmentation of Kaempferol –acetyl-glucoside.

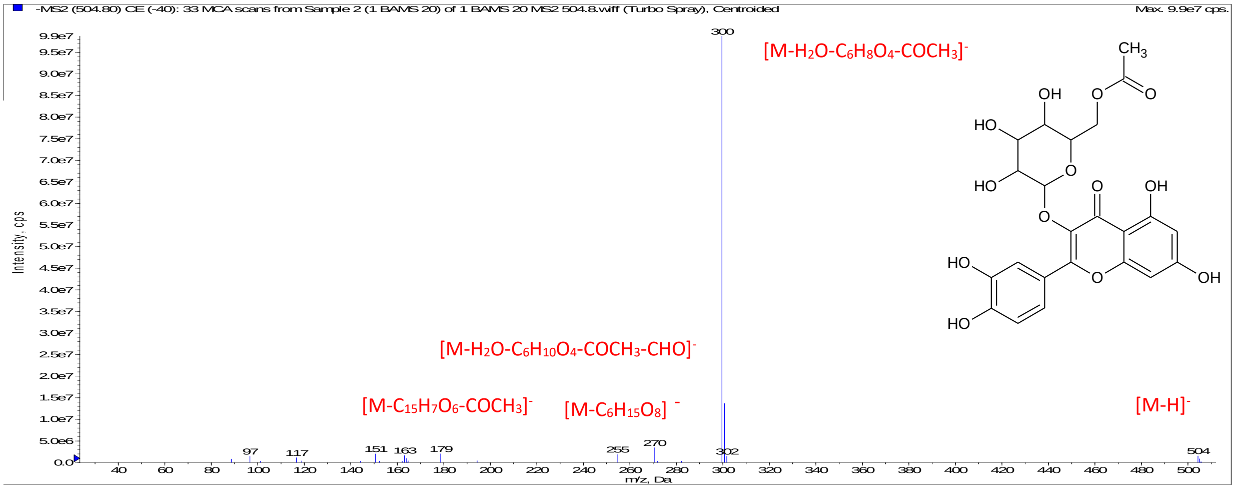

Figure 16. MS^2 fragmentation of Quercetin-acetyl-glucoside.

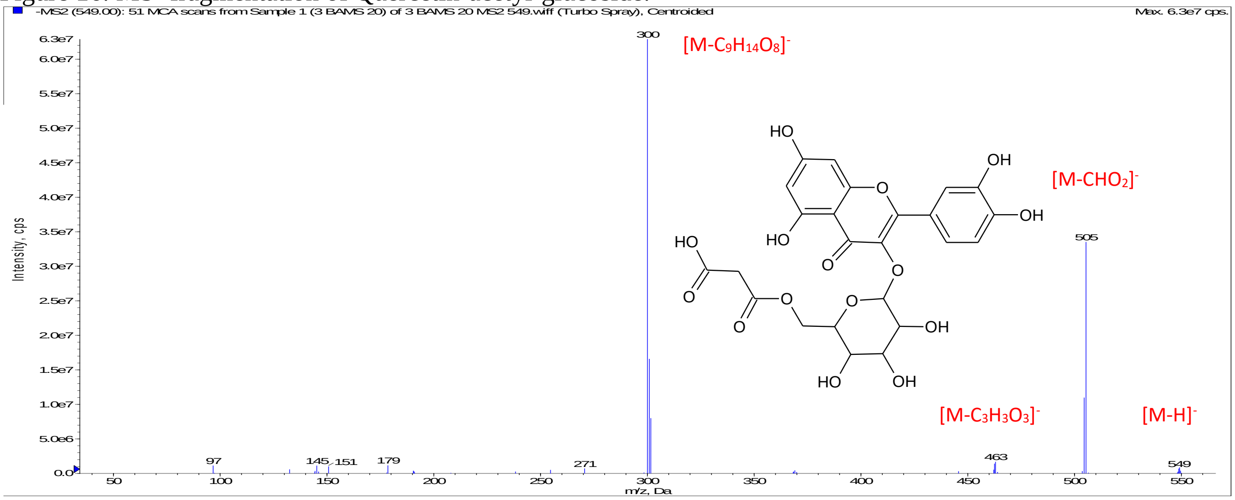

Figure 17. MS^2 fragmentation of quercetin-3-*O*-malonylhexoside.

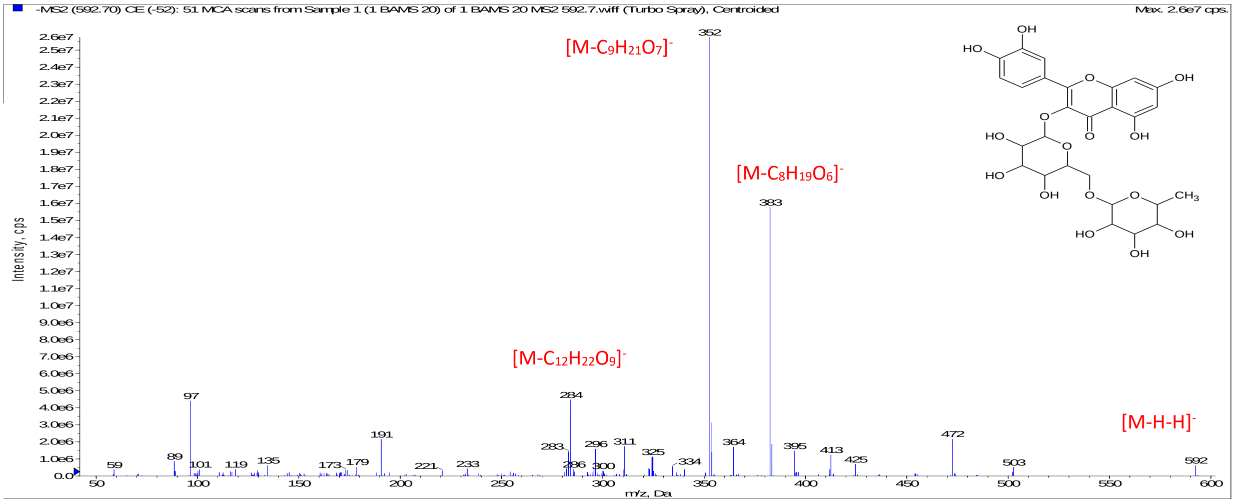

Figure 18. MS^2 fragmentation of Kaempferol -3- rutinoside.

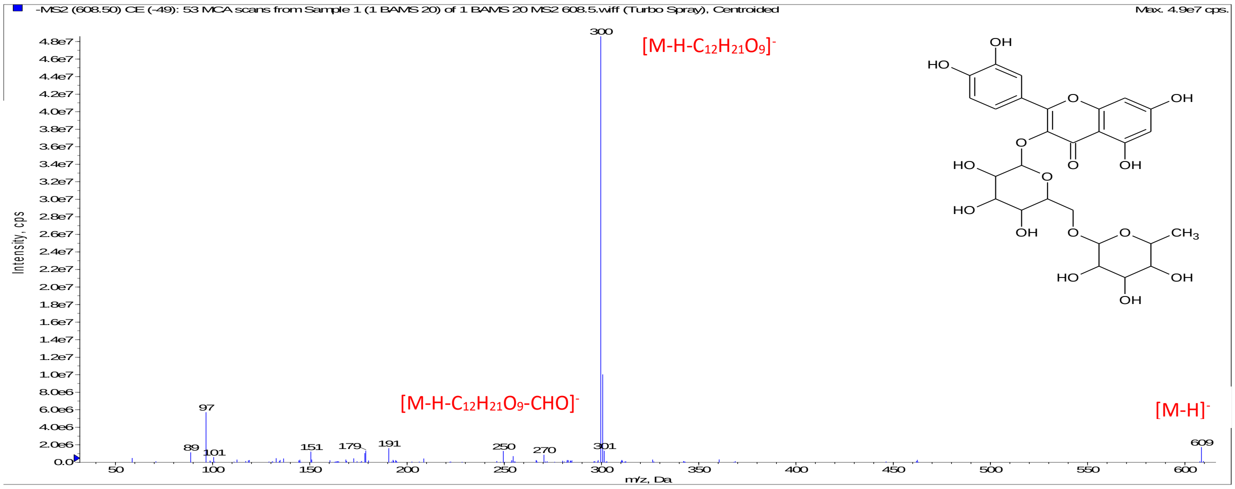

Figure 19. MS^2 fragmentation of Rutin.
